# ROS 2-Based Architecture for Autonomous Driving Systems: Design and Implementation

**DOI:** 10.3390/s26020463

**Published:** 2026-01-10

**Authors:** Andrea Bonci, Federico Brunella, Matteo Colletta, Alessandro Di Biase, Aldo Franco Dragoni, Angjelo Libofsha

**Affiliations:** 1 Department of Information Engineering, Marche Polytechnic University, 60131 Ancona, Italy; s1124068@studenti.univpm.it (F.B.); s1126127@studenti.univpm.it (M.C.); a.f.dragoni@staff.univpm.it (A.F.D.); s1121805@studenti.univpm.it (A.L.); 2 Department of Electrical and Information Engineering, Polytechnic of Bari, 70126 Bari, Italy; a.dibiase8@phd.poliba.it

**Keywords:** autonomous vehicles, ROS 2, mobile robotics, Edge-AI, embedded systems

## Abstract

Interest in the adoption of autonomous vehicles (AVs) continues to grow. It is essential to design new software architectures that meet stringent real-time, safety, and scalability requirements while integrating heterogeneous hardware and software solutions from different vendors and developers. This paper presents a lightweight, modular, and scalable architecture grounded in Service-Oriented Architecture (SOA) principles and implemented in ROS 2 (Robot Operating System 2). The proposed design leverages ROS 2’s Data Distribution System-based Quality-of-Service model to provide reliable communication, structured lifecycle management, and fault containment across distributed compute nodes. The architecture is organized into Perception, Planning, and Control layers with decoupled sensor access paths to satisfy heterogeneous frequency and hardware constraints. The decision-making core follows an event-driven policy that prioritizes fresh updates without enforcing global synchronization, applying zero-order hold where inputs are not refreshed. The architecture was validated on a 1:10-scale autonomous vehicle operating on a city-like track. The test environment covered canonical urban scenarios (lane-keeping, obstacle avoidance, traffic-sign recognition, intersections, overtaking, parking, and pedestrian interaction), with absolute positioning provided by an indoor GPS (Global Positioning System) localization setup. This work shows that the end-to-end Perception–Planning pipeline consistently met worst-case deadlines, yielding deterministic behaviour even under stress. The proposed architecture can be deemed compliant with real-time application standards for our use case on the 1:10 test vehicle, providing a robust foundation for deployment and further refinement.

## 1. Introduction

AVs are attracting growing interest in all areas of modern life, and interest in AVs has grown significantly in recent years. In 2025, the AV market underwent significant expansion, reaching a value of approximately 80.49 billion dollars [1]. This expansion is driven by several factors, including an increasing demand for safer and more autonomous driving solutions. The integration of AVs into modern transprt systems is linked to the level of autonomy of these vehicles. A key factor is the SAE J3016 Society of Automotive Engineers classification system [2], which defines six distinct levels of vehicle automation, from Level 0 (no automation) to Level 5 (full automation). In Europe and many other countries, Level 3 autonomous driving technology (Conditional Automation: the vehicle handles most of the driving tasks, but the driver must remain ready to take control if required) is currently permitted within defined speed limits. For autonomy levels 1 and 2, four-wheel commercial vehicles are already required to adopt autonomous technologies [3] such as advanced driver assistance systems (ADAS) [4,5], i.e., automatic emergency braking (AEB), driver drowsiness monitoring (DMS) and many others. For two-wheel vehicles, there is no specific regulatory requirement yet, but these technologies will be introduced gradually. In some high-end models they are optional. The delay can be attributed to increased caution due to the complexity of the dynamic behaviour of two-wheelers [6,7]. Given the rapid evolution of autonomy levels and future demands for autonomy in other automotive applications, the development of flexible software (SW) architectures for AVs is essential to design lightweight and scalable platforms that facilitate and promote the growth of autonomy in various mobility fields.

This paper proposes a ROS 2 architecture to design and develop an open-source, lightweight, scalable, and adaptable platform to facilitate the development and functional testing of autonomous vehicles.

This is accomplished through the adoption of a layered architecture built around a rule-based decision tree (RBDT) and a finite state machine (FSM). The RBDT is chosen for its transparency and deterministic behaviour, enabling clear interpretation of sensor inputs and context with minimal computational cost; meanwhile, the FSM is adopted to ensure predictable transitions between driving behaviours, supporting scalability and ease of maintenance through its modular structure. The proposed design can be easily extended to incorporate additional inputs and can be adapted to different vehicles and application scenarios, thus broadening its applicability to the wider class of terrestrial mobile robots.

The remainder of this paper is organised as follows: Section 2 reviews related works and places the present study within the existing literature. Section 3 presents an overview of ROS2. In Section 4, the proposed system architecture is introduced and detailed. Section 5 describes the hybrid decision-making module, which integrates a rule-based decision tree with a finite-state machine. Section 6 outlines the experimental setup. Section 7 concludes the paper and summarises the main findings. Lastly, Section 8 discusses the planned developments and future directions of this work.

## 2. Related Works

This section reviews the most relevant studies and industrial frameworks that have influenced the design of modern autonomous driving architectures over a period of time that spans approximately from 2016 to 2025. The analysis includes both academic and industrial contributions addressing the evolution from traditional signal-oriented systems towards modular, service-oriented paradigms. Specifically, we discuss (i) the transition from signal-oriented Electronic Control Units (ECUs) to Service-Oriented Architectures (SOAs), (ii) the evolution of AUTOSAR from Classic to Adaptive platforms, (iii) the WATERS Industrial Challenge 2019 as a benchmark for heterogeneous real-time systems, and (iv) open-source ROS 2-based stacks, with a focus on Autoware as a representative example of end-to-end autonomous driving software.

### 2.1. From Signal-Oriented ECUs to Service-Oriented Architectures

Traditional automotive software has relied on signal-oriented communication among numerous Electronic Control Units interconnected through in-vehicle networks (e.g., CAN/FlexRay). Although this approach has historically ensured robustness, exhibits well-documented limitations in scalability, flexibility, and maintainability as modern vehicles integrate large numbers of ECUs and exchange increasing data volumes [8,9]. These constraints have motivated a progressive transition towards SOAs, in which functions are encapsulated as interoperable services communicating via standardised protocols (e.g., SOME/IP, DDS, gRPC, REST). SOAs foster modularity, reuse, and high-bandwidth data exchange across heterogeneous platforms [10,11].

This hardware evolution has profound implications for the design of software architecture. Modern in-vehicle systems must now support distributed computation across multiple processing units, flexible deployment of services, and seamless interoperability among components developed by different vendors. Consequently, the software layer must adopt modular, service-oriented principles that decouple functionality from specific hardware nodes, ensuring scalability and ease of integration. In this context, middleware such as ROS 2 [12], with its DDS-based communication model and fine-grained Quality of Service policies, provides an effective foundation for implementing these service-oriented paradigms within autonomous vehicle architectures.

### 2.2. AUTOSAR Classic and Adaptive for Automated Driving

Within the automotive domain, the AUTOSAR (AUTomotive Open System Architecture) standard has established itself as the de facto reference framework for in-vehicle software. Its goal is to provide a common architecture that enables interoperability, reusability, and safety between heterogeneous Electronic Control Units (ECUs) developed by different suppliers. Over time, AUTOSAR has evolved into two complementary platforms—Classic and Adaptive—each designed to meet different system requirements and different hardware objectives [10,11].

Classic AUTOSAR was designed for resource-constrained ECUs and is used primarily in real-time safety-critical applications, such as braking, powertrain, or steering control. It follows a signal-oriented communication paradigm, where software components (SWCs) exchange predefined signals over static networks such as CAN, LIN, or FlexRay. This design ensures deterministic timing, high reliability, and compliance with functional-safety standards (ISO 26262 [13]), but limits flexibility: all tasks, communication patterns, and scheduling parameters are fixed at compile time. Classic AUTOSAR typically runs on small microcontrollers under the proprietary AUTOSAR OS, it emphasises static configurations and predictability over dynamic adaptability.

With the growing computational demand of Advanced Driver Assistance Systems (ADAS) and autonomous driving functions, the rigidity of the Classic platform has become a limiting factor. To overcome these constraints, the Adaptive AUTOSAR platform was introduced as its natural evolution, targeting high-performance computing units such as automotive-grade SoCs, multi-core CPUs, and GPUs. Adaptive AUTOSAR shifts from a signal-oriented to a service-oriented model, where applications expose and consume services dynamically through standardised communication frameworks such as SOME/IP or DDS over Automotive Ethernet. This enables runtime discovery, dynamic deployment, and over-the-air (OTA) software updates—features essential for modern connected and autonomous vehicles.

Furthermore, Adaptive AUTOSAR runs on POSIX-compliant operating systems (e.g., Linux, QNX, Integrity), allowing multi-process execution, high-bandwidth data exchange, and integration with AI-based and perception-heavy workloads. It also incorporates native support for cybersecurity (ISO/SAE 21434 [14]) and system diagnosability, expanding the scope beyond deterministic safety to include reliability and security in connected environments. In practice, modern vehicles increasingly combine both standards: Classic AUTOSAR handles deterministic control loops and safety-critical functions, while Adaptive AUTOSAR manages perception, planning, and communication-intensive tasks, providing scalability and long-term updatability for autonomous driving architectures.

Although AUTOSAR represents the industrial reference framework, we propose a custom ROS 2 based architecture for the following reasons. AUTOSAR is primarily designed for production-grade ECUs operating under strict certification. In contrast, our proposed architecture targets research and prototyping environments where flexibility, modularity, and rapid iteration are essential. AUTOSAR’s static configuration model and its dependency on proprietary toolchains hinder adaptability to heterogeneous hardware platforms such as embedded GPU boards (e.g., NVIDIA Jetson), which are increasingly adopted in experimental AV development. Therefore, the proposed ROS 2-based design provides a lighter, open-source, and dynamically reconfigurable alternative, while retaining service-oriented principles consistent with Adaptive AUTOSAR but optimised for scalability, distributed computing, and rapid functional testing.

### 2.3. ROS 2-Based Open-Source Stacks: Autoware as a Prominent Example

Open-source full-stack platforms have substantially increased the availability for academic and industrial research on autonomous driving by providing reusable modules, tooling, and validation infrastructure. Among these, Autoware has gained wide adoption as a modular end-to-end stack originally built on ROS and subsequently evolved to ROS 2 to improve middleware performance and distributed operation [15]. Autoware implements the canonical pipeline—localisation, perception, prediction, planning, and control—on top of a publish/subscribe middleware, with extensive support for sensing (LiDAR, camera, GNSS/IMU), high-definition maps, and simulation/visualisation tools.

On the perception side, it combines classical and learning-based methods, such as NDT-based localisation, clustering, and model-based detection, as well as deep architectures such as PointPillars and YOLO families [16]. It also integrates sensor-fusion filtering (e.g., EKF/UKF) for reliable tracking and object association across heterogeneous sensors.

The planning subsystem encompasses global route generation and local motion planning (e.g., lattice, A*, OpenPlanner), while the control stack provides several trajectory-follower algorithms, including Pure Pursuit and Model Predictive Control (MPC) [17].

In addition to the software modules themselves, contributions from the Autoware ecosystem-community, documented interfaces, training programme, and widespread deployment across multiple vehicles have been instrumental in disseminating best practices and accelerating technology transfer from research to prototyping and pilots. Reports highlight its use across countries, companies, universities, and multiple platforms, underscoring its portability and extensibility. More recently, a comparative analysis with another open-source autonomous driving software platform, Apollo 10.0 [18], highlighted how migration to ROS 2 enables deterministic DDS-based communication and improved support for distributed implementations across multiple compute nodes, essential for modern AV architectures.

Despite its maturity and comprehensive design, Autoware targets high-performance computing platforms and production-grade vehicles, where hardware resources and deployment infrastructure are not constrained. Its modular stack comprises a large number of interdependent nodes, GPU-accelerated perception pipelines, and complex configuration layers, which make it less suitable for small-scale or resource-limited experimental setups. In contrast, the ROS 2-based architecture proposed in this work was conceived as a lightweight research-oriented framework enabling rapid prototyping, real-time validation and deployment on embedded platforms such as NVIDIA Jetson or STM32 microcontrollers. This approach complements the Autoware ecosystem by focus on modularity and simplicity, bridging the gap between academic experimentation and full-scale autonomous driving frameworks.

## 3. Overview on ROS 2 for Autonomous Driving

ROS 2 has become a key middleware for building complex and distributed autonomous systems in both robotics and automotive domains. A recent survey provides a detailed overview of its contribution to robot autonomy [12]. As the natural evolution of ROS, ROS 2 was designed to overcome the main limitations of its predecessor, particularly in terms of real-time communication, scalability, reliability, and cross-platform interoperability. These improvements make it suitable for automotive applications, which require reliable data exchange, modularity, and deterministic behaviour to ensure safe and predictable system operation. The following are the most important features of ROS 2.

**A bridge between Research-Oriented and Production-Grade architectures**

The landscape of autonomous driving architectures spans from production-grade industrial systems to research-oriented experimental platforms. Production systems such as Tesla’s FSD, Huawei’s ADS, and Baidu’s Apollo represent highly mature frameworks designed for deployment on full-scale vehicles with extensive sensor suites, high-performance computing clusters, and substantial engineering resources. While these architectures demonstrate state-of-the-art performance in real-world conditions, their complexity, hardware requirements, and often proprietary nature limit accessibility for academic research and rapid prototyping.

In contrast, the ROS 2-based architecture proposed in this work, targets research-oriented prototyping and validation on resource-constrained embedded platforms. The proposed framework is explicitly designed to operate on affordable computing units such as NVIDIA Jetson Orin Nano (NVIDIA Corporation, Santa Clara, CA, USA) or Raspberry Pi 5 (Raspberry Pi Ltd., Cambridge, UK), enabling rapid iteration without the infrastructure overhead typical of production systems. The architecture’s lightweight modularity allows seamless integration of new perception algorithms, planning strategies, or sensor modalities with minimal refactoring, supporting incremental development cycles essential for experimental research. Furthermore, the 1:10 scale vehicle platform enables controlled experimentation with advanced state-of-the-art techniques in a safe, repeatable indoor environment, allowing researchers to validate novel algorithms before committing to full-scale deployment. The architecture thus complements production-grade frameworks by providing a research-enabling platform optimized for agility, transparency, and resource efficiency.

**Communication Model and Dynamic Discovery**

ROS 2 adopts a decentralized publish/subscribe communication paradigm in which independent software components, called nodes, exchange messages over typed topics. This modular architecture simplifies development, testing, integration, and accelerates prototyping across distributed hardware platforms. The adoption of the Data Distribution Service (DDS) as the underlying middleware enables automatic discovery of publishers and subscribers at runtime, allowing communication links to be established dynamically without prior configuration. Unlike traditional static architectures, where data exchanges must be explicitly defined in advance, DDS provides self-configuring connectivity that adapts as nodes join or leave the network, significantly enhancing flexibility and robustness in distributed systems.

**Real-Time Systems: Definition and Requirements**

A real-time autonomous-vehicle system is a computing system whose correctness is defined jointly by functional results and adherence to bounded temporal constraints along the perception, prediction/planning and control chain [19,20].

These constraints are specified as end-to-end timing properties (notably maximum reaction time (input-to-actuation) and maximum data age (information freshness)) and must be met predictably under the system operating conditions [21].

In modern practice, perception/prediction is commonly modelled as soft real-time, whereas closed-loop vehicle actuation remains hard real-time. In hard real-time, any missed deadline is a system failure, since timing constraints are part of the system’s requirements (e.g., closed-loop actuation for braking/steering). In contrast, soft real-time allows for occasional misses with graceful Quality of Service (QoS) degradation.

In this work, a 100 ms end-to-end reaction time budget is adopted as a design constraint for human-level safety. Zhao et al. [22] demonstrate on industrial Level-4 fleets that the perception–decision pipeline must keep total latency below 100 ms for the vehicle to react faster than a human driver. Accordingly, this bound is used to allocate timing budgets along the perception–planning chain.

**Quality of Service and Real-Time Execution**

A core strength of ROS 2 is its extensive exposure to DDS QoS policies, which allows for precise tuning of communication characteristics such as latency, reliability, durability, history depth and liveliness. Fine-grained control over these parameters supports heterogeneous data streams, for example low-latency channels for sensor telemetry and highly reliable channels for safety-critical actuation commands. When these QoS mechanisms are paired with a real-time Linux kernel, ROS 2 can sustain control loops that satisfy stringent temporal constraints. Empirical results report bounded end-to-end latencies well below 500 μs even in distributed deployments, underscoring the feasibility of ROS 2 for safety-critical vehicle control [23]. Furthermore, the integration of the PREEMPT_RT patch transforms the standard Linux scheduler into a fully preemptible one, reducing worst-case latencies and end-to-end jitter by an order of magnitude and enabling ROS 2 to meet the deterministic performance targets typical of modern autonomous driving controller [24].

**Portability, Composition, and Lifecycle Management**

The same middleware abstractions that facilitate determinism also decouple application logic from hardware specifics. Standardized ROS 2 message types and interfaces insulate software components from vendor-dependent characteristics of sensors and actuators, thus improving portability across heterogeneous compute nodes (e.g., x86/ARM, CPU/GPU, SBC/edge). ROS 2 additionally provides a lifecycle management model and component containers that enable controlled startup/shutdown, fault containment, and in-process composition to minimize overhead and jitter, which are key factors for stable operation in resource-constrained, real-time settings

**Security**

Cybersecurity is a first-order concern for in-vehicle and V2X communication. ROS 2 supports DDS-Security plugins that implement a public key infrastructure (PKI) based on X.509 certificates, offering end-to-end encryption, mutual authentication, and fine-grained access control at the level of topics, services, and actions. These mechanisms strengthen the communication fabric against contemporary cyber threats and are particularly relevant for safety-critical autonomous platforms [25].

**Tooling and Ecosystem**

ROS 2 takes advantage of a mature toolchain tailored for robotic system development. Visualization (e.g., RViz), data recording and replay (ros2 bag), and simulation environments (e.g., Gazebo) streamline debugging, validation, and continuous integration/continuous deployment workflows. This ecosystem reduces development time and enhances system reliability and maintainability by enabling hardware-in-the-loop (HIL) and software-in-the-loop (SIL) testing at scale [12].

**Implications for Autonomous Vehicles**

Overall, these features position ROS 2 as a solid, modular and scalable foundation for autonomous driving. Developers can distribute perception and planning pipelines across multiple compute nodes, tailor QoS profiles to the latency and reliability needs of diverse sensors and actuators, reuse components across hardware variants, and integrate security by design. Consequently, ROS 2 provides a compelling middleware choice that supports both rapid prototyping and production-oriented implementations of autonomous vehicle architectures.

## 4. The Proposed ROS 2-Based Architecture for Autonomous Driving

In this section, the preliminary modular composition of the proposed architecture is presented. It is designed to ensure the basic operation of a self-driving vehicle, that is, to analyse its environment, understand, and interpret it accurately, and then perform the manoeuvres necessary to navigate it safely and effectively. The following sections address the main modules.

### 4.1. General Overview of the Architecture

**Figure 1:**
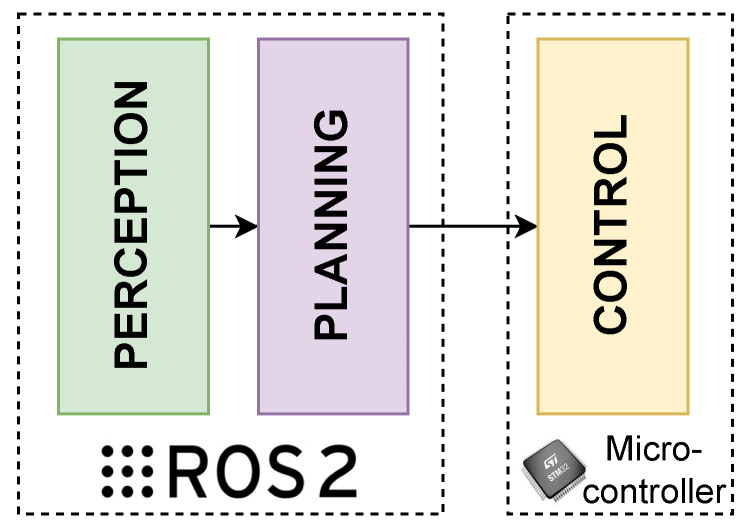
General overview of the proposed layered architecture.

Referring to Figure 1, the proposed general architecture is organised into three main layers: perception, planning, and control.

Concerning the three layers, the ROS 2 framework implements only the two highest layers, namely perception and planning. The control layer is at a lower level and is responsible for motion control of vehicle’s actuators and for the execution of commands planned by the higher-level modules. It interfaces with the output of the planning layer which provides the necessary set points to the controllers, as well as with the sensor domain of the perception layer to obtain the necessary feedback for motion control. An application provides an example of a motion control level for AVs [26], where a PID control of both longitudinal and lateral dynamics is proposed and implemented. At the same level, it is possible to execute nonlinear or linear control algorithms that perform better than PID, even ensuring tight execution times [27].

**Figure 2:**
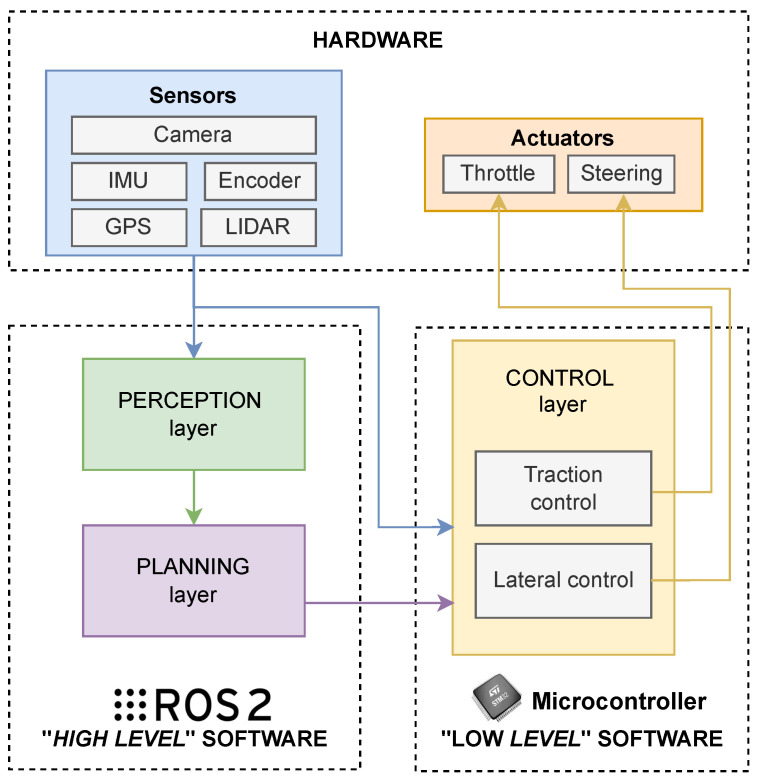
A detailed representation of the interactions among the layers of the proposed architecture.

Figure 2 further details the interactions among layers and introduces the vehicle’s hardware layer, with which the architecture must interface. It is divided into two blocks that distinguish between the sensor domain (Sensors module) and the actuator domain (Actuators module). The listed sensors represent a selection of devices commonly integrated into autonomous vehicles, while the actuator domain generally presents the throttle and steering control of a vehicle. The Perception layer interfaces with the sensor domain, handling data acquisition from all sensors installed on robotic or autonomous platform, as well as their preprocessing and information extraction. For instance, some activities carried out in this context are object detection and the analysis of depth data to cluster and identify potential obstacles. This layer is also responsible for distributing the information extracted from the sensors to all other planning components within the architecture (see Figure 2). The Planning layer handles the calculation of the vehicle trajectory based on environmental information extracted and provided by the Perception layer. Indeed, the Planning layer generates the trajectory, for example, by implementing obstacle avoidance strategies using obstacles position information. Subsequently, the reference trajectory is decomposed in speed and orientation, and the respective set points are provided to the Control layer, so that the system is correctly guided along the desired path (see Figure 2).

It becomes evident that both the Perception and Control layers require access to sensor data. However, we propose to design the architecture with decoupled sensor readings for the two layers. In fact, the Control layer does not access sensor data through the Perception layer but instead interfaces directly with the sensor domain. This design choice is motivated by the fact that the Control layer and the Planning layer are implemented on different hardware and software platforms (a microcontroller based on C/C++ for the Control layer and an embedded computing platform with ROS 2 for the Perception layer). In addition, the two layers require access to different sensor data streams operating at different frequencies, e.g., Table 1 presents several sensors accessed by both Perception and Control layers, highlighting the different data stream frequencies.

The architecture was divided into the proposed layers to improve the system reliability, organization and maintainability, by isolating functions and simplifying debugging. This structure also enables efficient management of large sensor data volumes such as those from LiDARs (Light Detection and Ranging), Cameras, and IMU (Inertial Measurement Unit), reducing latency and improving processing speed through tailored data handling techniques. Finally, layers separation allows for easier scalability and adaptability of the system, as each layer can be optimized and modified independently. This architecture is general enough to be adapted to different autonomous systems, even more challenging than four-wheelers [28,29], such as autonomous robotic systems [30,31] or advanced two-wheelers [32].

### 4.2. The Control Layer Architecture

The low-level control of the three-tier architecture (Perception–Planning–Control) was built on the model-free solution proposed in [26]. It allows for longitudinal and lateral dynamics control on a 1:10 scale car platform. However, the architecture is designed to allow the proposed controller to be replaced with other, even more sophisticated, controllers that work on the same inputs and outputs. The planned trajectory is parametrized in terms of curvature and vehicle speed. The planner should provide a speed reference $v_{\mathrm{ref}}$ and a curvature reference $\kappa_{\mathrm{ref}}$, then the desired vehicle’s yaw rate is easily calculated as ${\dot{\psi }}_{\mathrm{ref}}=v_{\mathrm{ref}}\;\kappa_{\mathrm{ref}}$ as proposed in [26].

**Figure 3:**
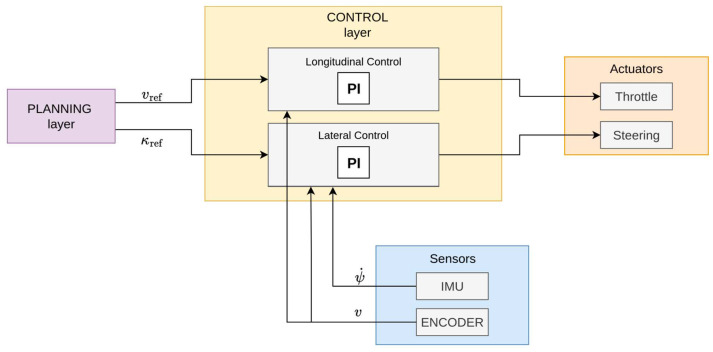
Block diagram of the control layer, detailing the configuration of the longitudinal and lateral PI controllers driven by $v_{\mathrm{ref}}$ and $\kappa_{\mathrm{ref}}$ provided by the Planning layer.

The overall control layer consists of two simple decoupled PI controllers shown in Figure 3:

- a longitudinal PI controller regulates the traction motor PWM duty cycle to track the speed reference using encoder feedback;
- a lateral PI controller that commands the steering servo angle to track the yaw-rate reference using IMU-based yaw-rate measurements.

Both controllers are implemented in discrete time with a 10 ms sampling rate using the standard parallel PI form and a conditional-integration anti-windup scheme to handle actuator saturation due to steering angle and motor voltage limits. The design is model-free and does not require the integration of vehicle dynamics models. It relies only on onboard sensors, such as the DC motor quadrature encoder for estimating vehicle’s speed and the IMU sensor that provides the yaw rate measure. The gains of the controller were empirically tuned to achieve an acceptable rise time, overshoot, and steady-state error for both speed and yaw-rate loops.

### 4.3. Detailed Overview of the ROS2 Architecture for Perception and Planning Layers

**Figure 4:**
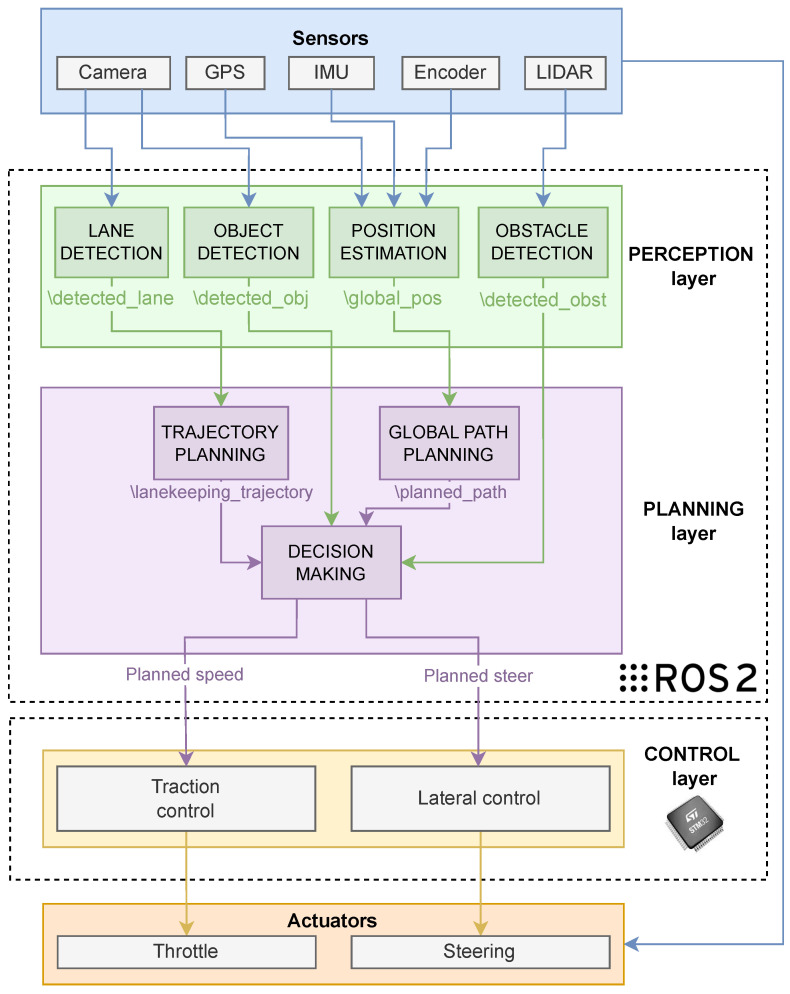
Proposed detailed architecture for Autonomous Vehicles.

As introduced in Section 4.1, the Perception and Planning layers represent the highest-level components of the architecture developed in ROS 2. A detailed description of their design is provided in Figure 4. In the diagram, the blocks within the ROS 2 architectural layers correspond to ROS 2 packages, each containing the necessary nodes to implement the specific functionalities of the package. The arrows that originate from the package blocks illustrate the communication between packages: specifically, the block from which an arrow originates represents the publisher of a given topic, while the block that receives the arrow represents the subscribing package.

The Perception layer, as detailed in Figure 4, delineates the essential set of packages required to interface with and process data originating from the sensors commonly integrated into autonomous vehicles, represented within the *Sensors* block of the diagram. Each package is specifically designed to interface with the sensors from which it requires data, and it analyses and processes them in order to provide information, that can be interpreted by the decision-making system, to the Planning layer. At the algorithmic level, the perception modules were integrated using approaches tailored to resource-constrained embedded platforms, i.e., suitable for deployment under limited on-board computational resources. The object detection module employs a custom YOLO11 model optimized for embedded deployment through reduced-precision inference (FP16), balancing detection accuracy with computational efficiency on resource-constrained hardware. The lane detection pipeline implements the RANSAC (RANdom SAmple Consensus) method for robust line fitting in the presence of outliers, coupled with an adaptive pure pursuit controller that dynamically adjusts the look-ahead distance based on vehicle velocity and lane curvature. For obstacle detection, the system utilizes DBSCAN (Density-Based Spatial Clustering of Applications with Noise) clustering algorithm, which groups nearby point cloud detections within a distance threshold ϵ that satisfy a minimum density requirement (MinPts neighbors). This density-based approach effectively identifies spatially coherent obstacle regions while automatically rejecting spurious sensor returns and low-density noise as outliers, enhancing the robustness of the perception pipeline in dynamic environments. A crucial aspect of this design is the introduction of custom ROS 2 message types for inter-node exchanges. By defining custom messages as shown in Table 2, the system gains finer control over the information transmitted aggregating similar data in a single message. Thus, the planning node can publish only what is strictly necessary to execute a manoeuvre (e.g., a single “ObstacleDetected” message encompassing type of obstacle, its direction and distance). This approach not only streamlines communication overhead but also improves maintainability, since each message type is self-documenting with semantically clear fields and the resulting architecture has less complexity regarding topic count since they can be compressed.

The planning layer is composed of 3 main packages: “trajectory planning” and “global path planning” are necessary to further interpret and pre-process information from the perception layer, while “decision making” is the main package of this layer.

The architecture relies on the Decision Making (DM) package, a central component in the Planning layer that receives key input data (e.g., perception outputs, sensor measurements and high-level commands) via ROS 2 topics. Conceptually, this results in a funnel-like decision structure: instead of distributing the responsibility for data aggregation across multiple modules, the DM acts as the primary repository for incoming messages and as the sole entry point to the decision pipeline.

**Figure 5:**
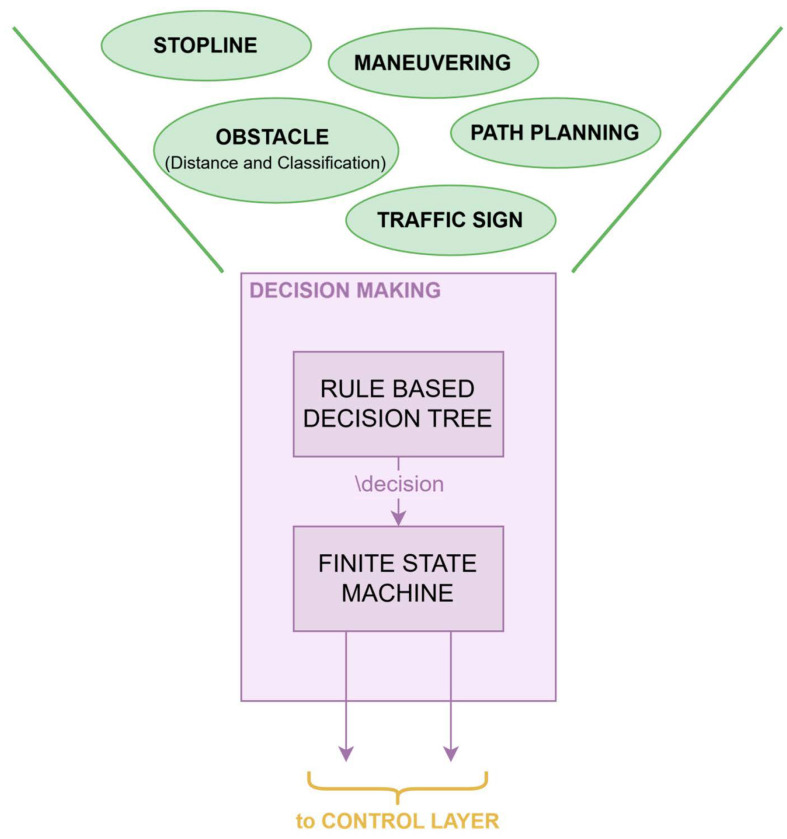
Detailed overview of the Decision Making package.

A more detailed structure of the DM package is shown in Figure 5. All the different inputs are processed by a rule-based decision tree and collapsed into a single symbolic decision, which is then interpreted by the finite-state machine to generate the planned speed and steering angle for the Control layer. In this way, the DM package always maintains a coherent snapshot of the vehicle state and surrounding environment, from which it derives appropriate control outputs (e.g., target velocity and curvature) and publishes them toward downstream controllers.

This centralisation provides clear benefits in terms of integration and maintainability: new sensors or complementary algorithms can be attached to the same funnel node, and their outputs immediately become available to the decision logic without introducing additional arbitrators. However, concentrating all decision-relevant streams into a single package also introduces a clearly identifiable single point of failure and a potential performance bottleneck. On high-performance platforms, this limitation is often addressed by distributing decision logic across manoeuvre-specific nodes, by introducing hierarchical arbitration layers, or by adopting shared blackboard architectures maintained by dedicated fusion modules. Although these decentralised patterns generally improve fault isolation and scalability, they also involve higher coordination complexity, a larger number of ROS 2 entities, and more demanding verification effort, which are difficult to justify in the proposed small-scale experimental setup.

For this reason, this work intentionally proposes a funnel-based DM as a conscious architectural trade-off given the limited set of manoeuvres and sensors. The result is a single authoritative decision node that simplifies debugging, logging, and rapid prototyping. In addition, the event-driven execution and zero-order hold snapshot semantics described in Section 4.5 mitigate the risk of overload by avoiding redundant evaluations. Overall, the design should be interpreted as a deliberately centralised yet evolvable baseline, which can later be refactored towards the aforementioned decentralised patterns as system complexity and safety requirements increase.

The proposed architecture leverages ROS 2’s DDS-based QoS policies to tailor communication reliability and latency characteristics according to each data stream’s role in the perception–planning pipeline. QoS profiles are differentiated based on whether a topic serves visualization/debugging purposes or carries perception-critical information required for decision-making.

**Perception-critical data streams.**

Topics carrying preprocessed sensor data destined for perception algorithms (e.g., masked frames for lane detection, preprocessed images for object detection) are configured with RELIABLE delivery semantics, KeepLast(2) history depth, and VOLATILE durability. A deadline constraint in the range of 40–50 ms is enforced to ensure timely availability of fresh perception inputs. This configuration guarantees that downstream perception nodes receive complete frame sequences without message loss, which is essential to maintain detection consistency and prevent spurious maneuver triggers due to dropped observations.

**Visualization and debugging streams.**

Conversely, topics used solely for human operator monitoring or off-line inspection (e.g., raw camera frames, intermediate visualization outputs) adopt BEST_EFFORT reliability with KeepLast(1) history. This lightweight profile minimizes computational and network overhead when subscribers are active, while allowing frame drops under load without impacting the core perception–planning loop. The conditional publishing mechanism further reduces resource consumption by suppressing visualization outputs when no subscribers are present.

**Planning and control commands.**

High-level planning outputs (e.g., velocity and curvature references) are published with BEST_EFFORT semantics and minimal buffering (KeepLast(1)), reflecting the event-driven execution model of the Decision-Making Package. Since the DMP updates commands only upon new perception inputs and retains the previous control state via zero-order hold, strict message delivery guarantees are not required. This design minimises latency and avoids queuing delays in the planning–control interface.

**Default QoS for auxiliary topics.**

All remaining inter-node communication channels (e.g., internal coordination signals, non-critical status messages) retain ROS 2 default QoS settings. Given the event-driven architecture and the absence of high-frequency periodic polling, default profiles provide sufficient reliability and responsiveness without introducing unnecessary configuration complexity or resource overhead.

This QoS stratification ensures that computational and communication resources are allocated proportionally to each topic’s criticality, thereby supporting the real-time performance requirements validated in Section 6.4.

### 4.4. Synchronization for Multiple Perception Algorithms

In real-time systems, e.g., AVs or autonomous robots, specific algorithms require input from heterogeneous data sources which are operating at different update rates, thus it is essential to perform data synchronisation prior to processing to ensure compliance with the timing constraints. Unsynchronised data can describe inconsistent states of the vehicle and its surrounding environment, reducing the reliability of perceptions and planning modules. It is crucial, especially in real-time systems, to manage and minimise time delays between sensors operating at different frequencies.

**Figure 6:**
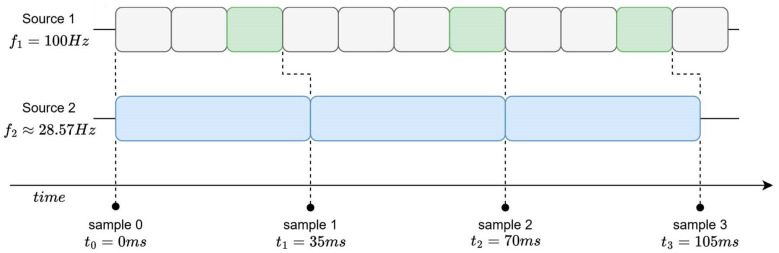
Downsampling—example of downsampling of Source 1 compared to Source 2.

**Figure 7:**
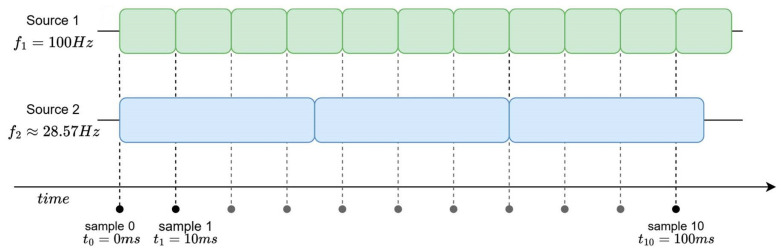
Upsampling—example of upsampling of Source 2 compared to Source 1.

From a software side, two complementary strategies exist: slow down the faster streams (downsampling, see e.g., Figure 6) or virtually speed up the slower ones (upsampling see e.g., Figure 7). Downsampling skips or filters portions of the faster streams, for example, by only using the most recent sample when slower data come in. Alternatively, complex approaches can be implemented to increase the success of recognising elements of interest [33].

Conversely, upsampling generates an output at the frequency of the fastest sensor by repeating the data from the slower sensors (a zero-order hold) or, in more complex applications, by estimating the missing data through interpolation techniques such as linear or spline interpolation. Some interpolation techniques that can be applied include classical First-Order Hold (FOH), also known as linear interpolation, as well as higher-order methods such as quadratic or cubic interpolation. In the specific context of temporal interpolation of video frames, more advanced approaches, such as optical flow interpolation, can be employed to estimate intermediate frames. General approaches have already been developed [34,35], but their applicability in real-time systems must be carefully evaluated, as the computational overhead of frame interpolation techniques may exceed the timing constraints of resource constrained platforms, making simple downsampling the more viable solution for video streams in such contexts.

**Figure 8:**
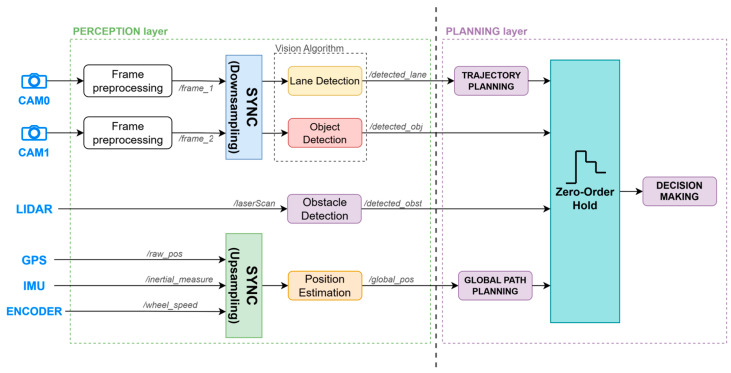
Block diagram highlighting the information flow between the Perception and Planning layers.

In the proposed architecture, shown in Figure 8, we adopt a hybrid synchronisation strategy tailored to the characteristics of each data stream. Downsampling is applied to high-frequency video streams from the cameras, where frame preprocessing modules synchronise the camera pair before feeding the perception algorithms (lane detection and object detection). For localisation data streams, specifically GPS, IMU, and encoder measurements, zero-order hold unsampling is employed to align these lower-frequency sensors with the system’s primary control loop rate. This approach maintains the most recent valid measurement until new data arrive, ensuring the continuous availability of localisation information without introducing artificial dynamics through interpolation.

The integration of these synchronisation techniques with the decision-making system, the planning layer, and the remaining architectural components is detailed in the following section.

### 4.5. Event-Driven Input Handling for the Decision Making Package

This section details the event-driven policy adopted to feed the Decision Making Package (DMP), which encapsulates the system decision logic. Instead of operating cyclically with a time-triggered loop, the DMP is recomputed only when at least one of its inputs publishes a new message. The sole inputs to the DMP, consistent with Figure 3, are trajectory_planning, object_detection, global_path_planning, and obstacle_detection. No explicit priority ordering is imposed: any new message from any of these inputs triggers an update. These streams are considered sufficient to activate the decision-making process because they encode information that can immediately alter manoeuvre selection (e.g., feasibility and geometry of the planned motion, collision risk, and salient objects or constraints in the scene).

**Motivation and benefits over cyclic computation.**

A time-triggered re-computation at a fixed rate can lead to redundant evaluations whenever the perception/planning state remains unchanged. In contrast, the event-driven strategy updates the DMP only upon the arrival of at least one new input, thereby avoiding unnecessary processing. This yields a lighter computational footprint, improves energy efficiency on embedded targets, and frees CPU cycles for perception and control tasks. If no input updates occur, the DMP retains the previous decision, avoiding unnecessary evaluations.

**Asynchronous inputs without explicit synchronisation.**

Unlike multi-sensor fusion pipelines that enforce strict barriers synchronisation, the DMP accepts input asynchronously. This removes the need for an exact time alignment between messages and avoids latency penalties and message drop typically associated with the synchronisation gates. As a consequence, the system remains responsive even when the involved publishers operate at different and time-varying rates (e.g., vision-derived detections versus lightweight geometric or planning cues).

**Implicit upsampling of non-updated inputs.**

Whenever a new message is received from any input node *i* at the *k*-th event, the snapshot evaluated by the DMP is constructed by combining the fresh input $u_{i,k}$ with the latest values available from all other nodes. Specifically, let $x_{i,k}$ denote the value associated with the input node *i* at the decision event *k*:

(1) $$x_{i,k}=\left\{\begin{array}{cc} u_{i,k} & \mathrm{if}\;\mathrm{input}\;i\;\mathrm{has}\;\mathrm{updated}\;\mathrm{at}\;\mathrm{the}\;k\text{-th}\;\mathrm{event},\\ x_{i,k-1} & \mathrm{otherwise}, \end{array}\right.$$

This formulation implements an implicit zero-order hold upsampling strategy, in which each input retains its last valid value until a new message is received. This mechanism preserves the temporal consistency in the DMP’s evaluation while maintaining the overall event-driven computational model. The general arrangement of the downsampling, upsampling (as discussed in Section 4.4) and the Zero-Order Hold mechanisms within the proposed architecture are shown in Figure 8.

## 5. Decision-Making Package: Rule-Based Tree and Finite State Machine Integration

The decision-making layer in the proposed architecture features a two-level modular design that separates high-level semantic interpretation of the environment from the assignment of the reference values that the vehicle should follow. However, this layer operates independently from the vehicle’s motion control layer, which handles sensor actuation and data acquisition. This structure improves robustness, maintainability, and scalability in autonomous navigation. Fully implemented within the ROS 2 ecosystem, the module consists of: (a) a rule-based decision tree and (b) a finite state machine, which will be integrated into a (c) hybrid decision making module.

### 5.1. Rule-Based Decision Tree—RBDT

The first stage of the adopted decision pipeline is a deterministically defined decision tree. For example, Chen et al. [36] observe that a decision tree has a clear structure, which visually represents the decision-making process, facilitating its understanding and interpretation.

A rule-based decision tree (RBDT) is chosen mainly because it provides predictability, the deterministic logic ensures that the system never exhibits unexpected or undesired behaviours. It is also highly interpretable, as each decision path in the tree can be directly traced and understood. Moreover, the computational efficiency of this approach makes inference extremely fast and thus suitable for embedded platforms.

The RBDT operates on a structured input vector consisting of seven discrete features derived from the perception and localisation layers. Table 3 provides the complete specification of the feature space, detailing the variables semantic role, data type, range, and the sensor modality responsible for its extraction.

This explicit enumeration establishes the Operational Design Domain (ODD) boundaries of the decision layer, ensuring that all inputs are traceable to physical sensor readings or validated map data. The feature set excludes raw sensor data that is continuously evaluated, relying instead on the symbolic abstractions produced by the RBDT node. This design choice improves interpretability and decouples decision logic from sensor-specific noise characteristics.

From this structured set of handcrafted rules (encoded as example feature vectors and target actions), the tree’s hierarchical structure is generated using the DecisionTreeClassifier from scikit-learn.

It is essential to clarify that the use of scikit-learn DecisionTreeClassifier does not constitute a conventional machine learning training process. Rather, the classifier serves as a rule compiler, transforming the handcrafted 416-scenario rule set into a tree structure optimised for efficient runtime evaluation. The data set is not collected from real-world observations, but is generated implementing a hierarchical decision function mapping input features to output actions according to predefined safety and traffic rules. The hierarchical rule structure ensures that safety-critical features (e.g., Obstacle) take precedence over navigation-related features (e.g., Sign, PathPlanning), effectively encoding domain expertise into a priority-ordered decision cascade. To preserve the original rule semantics without introducing data-driven distortions, the following constraints are enforced: no pruning is applied, as the tree must perfectly fit all authored examples (100% training accuracy is achieved and required, since the data set is noise-free by design); and concepts such as cross-validation, regularisation, or hyperparameter tuning are inapplicable, since the objective is exact rule encoding rather than empirical generalisation. In practice, the DecisionTreeClassifier is instantiated with default parameters, ensuring that the resulting tree is a faithful logical encoding of the input rules.

After the inference the RBDT outputs are symbolic commands (e.g., stop, lane_keeping, intersection…), which is then mapped to the corresponding state in a Finite State Machine.

A common concern in data-driven systems is their ability to generalise beyond the training set. However, this concept is largely irrelevant in the context of the proposed RBDT, as the system is not expected to extrapolate to unseen scenarios outside the authored ODD. The handcrafted nature of the rule set implies that: the tree encodes domain-specific knowledge rather than statistical correlations, eliminating the risk of spurious pattern recognition.

Among the possible FSM, we recommend YASMIN and Section 5.2 contains the reasons for this choice.

### 5.2. Finite State Machine—FSM

The execution of discrete behaviours selected by the decision layer is orchestrated by a Finite State Machine (FSM) implemented with YASMIN. The FSM models the vehicle behaviour as a set of macro-states (e.g., driving, stopping, intersection handling, overtaking, parking) connected by explicit transitions. These passages are triggered by high-level commands from the Rule-Based Decision Tree and by salient environmental events.

YASMIN provides native ROS 2 integration, a blackboard for data sharing between states, a web-based visualiser for runtime inspection, and support for hierarchical composition.

**Design properties.**

The FSM (a) enforces a clean separation between decision logic and action execution, (b) exposes an explicit and auditable transition structure, and (c) leverages shared data to keep states loosely coupled and reusable. This results in robust coordination of complex behaviours with bounded latency and straightforward monitoring during experiments.

**Scalability and extensibility.**

Extending the behaviour repertoire requires only the addition of a new macro-state and the registration of its transitions toward existing states, plus the mapping of the corresponding command in the RBDT. Because states interact through stable interfaces and shared data, extensions remain localised and do not affect the internal logic of other states. This property has proven essential for gradually incorporating new manoeuvres (e.g., additional intersection rules or specialised parking routines) without refactoring the overall execution pipeline.

### 5.3. Hybrid Decision-Making Module for Architectural Integration—HDMM

The proposed decision layer adopts a Hybrid Decision-Making Module (HDMM) that integrates an RBDT with a structured FSM to couple high-level intent selection with predictable execution. The RBDT abstracts perceptual and contextual evidence into symbolic commands (e.g., stop, overtake, intersection). These commands are mapped in a one-to-one fashion to FSM macro-states, each command uniquely selecting its corresponding state. The FSM then orchestrates the concrete behaviour through explicit transitions driven by environmental events and execution outcomes.

Using YASMIN’s hierarchical composition and shared-data blackboard, the module preserves a clean separation between *what* to execute (decision logic) and *how* it is executed (state logic and reference generation), thus ensuring traceability and bounded latency of the integration pattern under real-time constraints.

**Integration pattern.**

The HDMM operates as follows. The RBDT first publishes a symbolic action consistent with the current scenario; then the module activates the associated macro-state (in a one-to-one mapping) and configures the required references (e.g., velocity, curvature, manoeuvre parameters). In case of failure, or unknown output from the unexpected input data from the RBDT, there is also a fail-safe path to reach an *error* state, always available to ensure recovery.

**Benefits.**

- Separation of concerns: decision logic remains independent from execution details, simplifying validation and maintenance.
- Determinism and auditability: explicit transitions and state scoping provide predictable behaviour and an inspectable execution trace.
- Modularity and reuse: macro-states encapsulate self-contained behaviours that can be combined hierarchically.
- Scalability: extending the repertoire requires only adding a macro-state and registering its transitions, without refactoring existing state logic.

In practice, this Hybrid Decision-Making Module has proven effective for real-time coordination on the scaled vehicle platform, enabling consistent, safe behaviour across diverse driving scenarios while supporting incremental growth of the manoeuvrer set.

## 6. Experimental Setup and Results

To validate the proposed architecture, the system is deployed and tested on a physical platform 1:10 scale electric AV. It was initially developed to participate in the Bosch Future Mobility Challenge (BFMC), an international competition that promotes research and innovation in autonomous driving. The challenge required teams to develop algorithms using minimal hardware resources capable of navigating a 1:10 scale autonomous vehicle within a dynamic environment that replicates real-world urban scenarios.

### 6.1. Testing Environment

**Figure 9:**
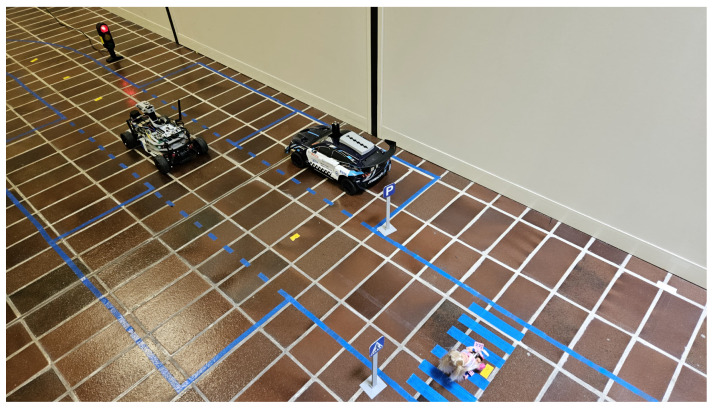
A section of the environment developed for validating the proposed architecture on the AV vehicle model. Since the floor is white between the different tiles, the most flexible and economical solution for marking out the lanes is to apply adhesive tape in a colour that can be easily filtered with colour masks, in our case blue.

A testing environment has been developed to replicate all the scenarios that the vehicle is expected to handle within a scaled city-like circuit that includes intersections, crosswalks, roundabouts, pedestrians, and parking areas. Figure 9 illustrates part of the reproduced environment.

The vehicle is expected to perform multiple tasks autonomously, including

- Lane keeping and trajectory tracking;
- Obstacle detection and collision avoidance;
- Traffic sign recognition;
- Intersection management;
- Dynamic overtaking and highway merging;
- Parallel parking;
- Pedestrian and crosswalk interaction.

A localisation system based on UWB (Ultra Wide Band) aims to simulate real-world scenarios in which GPS would be typically employed. This allows testing navigation in situations that are very close to realistic ones, such as global localisation, waypoint tracking, and trajectory planning based on absolute positioning. In our implementation, the UWB localisation system provides real-time position estimates with decimetre-level accuracy, which enables map-based navigation using a graph-based representation of the circuit.

### 6.2. Vehicle Platform and Hardware Architecture

**Figure 10:**
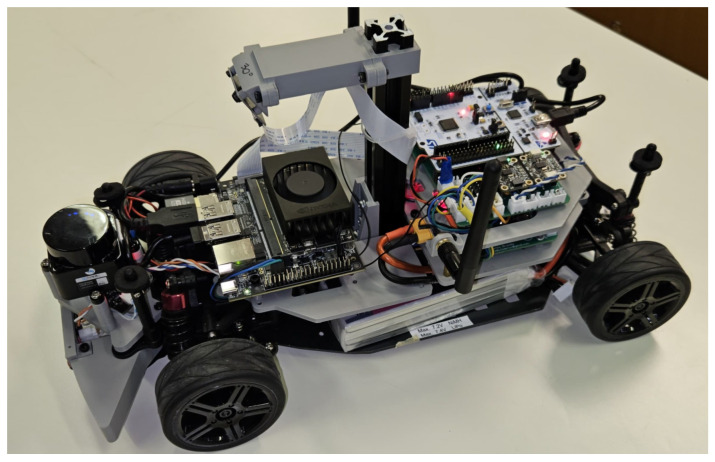
1:10 scale Vehicle Platform used for the implementation and validation of the proposed architecture.

The vehicle prototype adopts a layered mechanical architecture, as visible in Figure 10. The bottom layer houses the batteries and actuators, while the first layer accommodates the power distribution board in the rear section and the high-level computing unit in the front section. The second layer hosts the low-level controller board. A central vertical column serves as the support for the RGB camera. The LiDAR sensor is installed on the front section of the platform.

The two computing units are tailored to manage the computational requirements of the perception, planning, and control layers:

- A NVIDIA Jetson Orin Nano 8 GB (NVIDIA Corporation, Santa Clara, CA, USA) board serving as the high-level computing unit. This embedded GPU platform runs ROS 2 and manages all tasks related to the perception and planning layers of the proposed architecture.
- An STM32 Nucleo-F401RE microcontroller (STMicroelectronics, Geneva, Switzerland) is used for the low-level controller and sensors. It is responsible for motion control and sensor data acquisition at real-time rates.

The two units communicate through a UART serial interface, enabling reliable and efficient transmission of motion commands and feedback signals. Where required, to ensure greater reliability, it could be easily replaced by the CAN bus communication protocol which is widely used in automotive.

The state of the 1:10 scale vehicle is measured by the sensors reported in Table 4, and is driven by the actuators detailed in Table 5.

### 6.3. Architecture Scalability

The architecture’s modularity was demonstrated by its successful migration from an initial Raspberry Pi 5 platform to the more powerful NVIDIA Jetson Orin Nano to meet increased computational demands, highlighting its hardware scalability. The original processing unit chosen for the vehicle test platform was the Raspberry Pi 5, equipped with a Hailo-8L accelerator to handle object detection tasks. As the architecture grew in complexity, the Raspberry Pi 5 was no longer able to handle the growing processing demands. In particular, for tasks requiring more computationally intensive or real-time processing, the Raspberry Pi’s capabilities became inadequate. Thanks to its modular and multi-layered design, the proposed architecture was successfully migrated and implemented on a different hardware configuration consisting of NVIDIA Jetson ORIN Nano module. The software architecture was the same, and both platforms run Linux.

### 6.4. Real Time Performance Evaluation

To assess the performance of the proposed architecture, a worst-case execution time analysis approach was adopted, in line with the methodology discussed in [21]. Specifically, the Worst-Case Execution Time (WCET) was firstly evaluated for each complete perception-planning pipeline considered in this work. In addition to a component-level WCET characterisation, the evaluation was extended to a pipeline-level end-to-end timing analysis. This second level of analysis captures the cumulative latency experienced by the data as it traverses the architecture, from sensor acquisition and preprocessing through perception and planning, up to the generation of the control output. By combining WCET measurements with an end-to-end analysis of the main architectural pipelines, we provide a more comprehensive and system-oriented view of performance, enabling the identification of both local bottlenecks (node/callback level) and global bottlenecks (cross-node interaction and scheduling effects) that determine whether real-time constraints can be met under worst-case operating conditions.

Both analyses rely on timing data collected through LTTng (Linux Trace Toolkit Next Generation), a low-overhead tracing framework for Linux. In our context, LTTng is leveraged through ros2_tracing, a suite of multipurpose tracing tools for ROS 2 [37]. We use it to record timestamped tracepoints along the perception–planning pipeline execution, from which we derive both component-level WCET and end-to-end pipeline latency on real runs.

An LTTng trace is a time-ordered sequence of events, each tagged with a high-resolution timestamp, execution context (process/thread/CPU), and an event-specific payload. In ROS 2, the payload provides the identifiers needed to correlate callbacks and communications across nodes (e.g., executor transitions, callback and pub/sub handles, message metadata). The traces are post-processed to derive the target metrics.

**Worst-case execution time analysis**

Based on the metrics collected using the methodology described above, the worst-case end-to-end execution time of each perception–planning pipeline in the architecture (e.g., Vision, LiDAR, etc.) was computed.

Let Equation (2) denote the end-to-end execution time of a perception–planning pipeline in the architecture:

(2) $${\mathrm{WCET}}_{\mathrm{tot}}≜\sum_{\tau \in T}{\mathrm{WCET}}_{\tau }$$

where T is the set of tasks in the pipeline and $WCET_{\tau }$ is the WCET of the τ task of the pipeline.

The results of the acquisitions are reported in Table 6, which summarizes the timing measurements for each task composing the analyzed pipelines.

**Figure 11:**
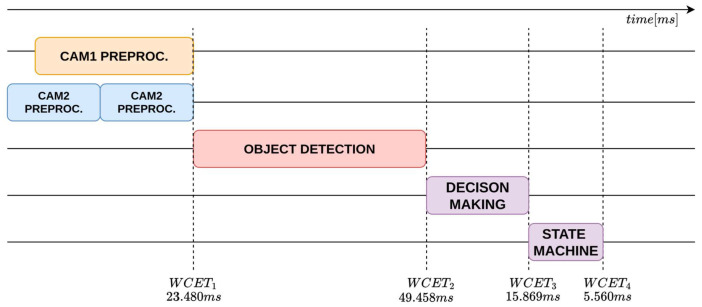
WCET analysis timeline of the object-detection perception–planning pipeline.

**Figure 12:**
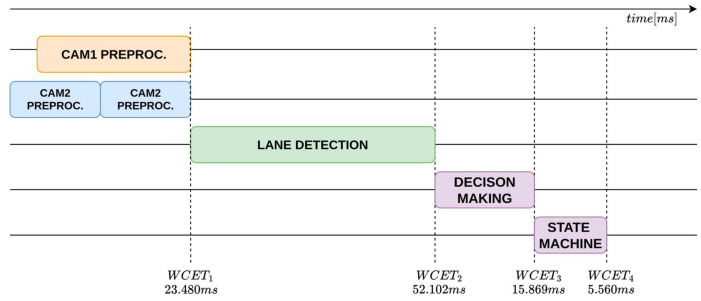
WCET analysis timeline of the lane-detection perception–planning pipeline.

**Figure 13:**
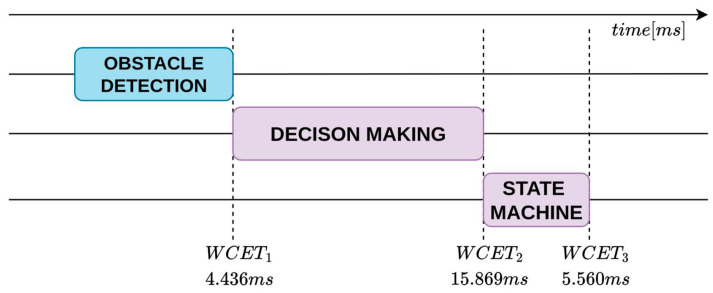
Timeline of the obstacle-detection perception-planning pipeline.

WCET measurements were collected in an isolated execution environment in which the entire proposed architecture was deployed and executed as a whole, while the system was otherwise free from unrelated workloads. During acquisition, compute, memory, and I/O resources were exclusively allocated to the architecture to eliminate external interference and obtain timing data representative of its execution. WCETs are evaluated for the main pipelines: object detection, to identify the type of obstacle; lane detection, to locate the lane boundaries around the vehicle; obstacle detection, to understand the distance from obstacles. These are shown respectively in the Figure 11, Figure 12 and Figure 13.

The analysis shows that the object-detection and lane-detection perception–planning pipelines exhibit the highest worst-case execution time (WCET) among the evaluated configurations. Indeed, based on the experimentally collected data, the total worst-case execution time of the object detection pipeline is $WCET_{\mathrm{tot}}=94.367\;\mathrm{ms}$, while the corresponding value for the lane-detection pipeline is $WCET_{\mathrm{tot}}=97.011\;\mathrm{ms}$. By contrast, the obstacle-detection pipeline achieves substantially lower WCET: $WCET_{\mathrm{tot}}=24.865\;\mathrm{ms}$.

**End-to-End execution time analysis**

Data were collected to allow an end-to-end timing analysis of the proposed perception–planning pipelines. This end-to-end analysis is crucial because it characterises the timing behaviour of the architecture as an integrated system, rather than as a set of isolated components as the WCET analysis does. The end-to-end analysis of the architecture accounts for ROS2 induced latencies across nodes, including message-passing overhead, DDS transport, executor dispatching, and callback queueing. As a result, the measured end-to-end latency reflects the effective responsiveness of the perception–planning loop under realistic execution semantics.

The Table 7 summarizes the results of the end-to-end timing analysis for the evaluated perception–planning pipelines.

**Figure 14:**
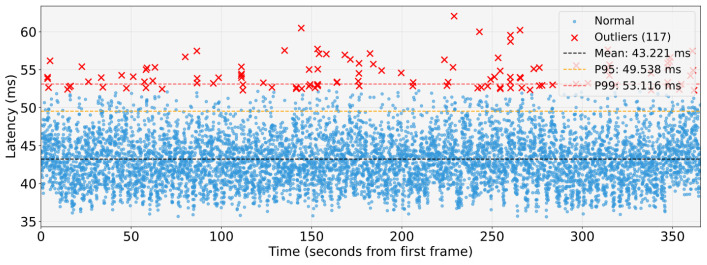
End-to-end timing analysis of the lane-detection perception-planning pipeline.

**Figure 15:**
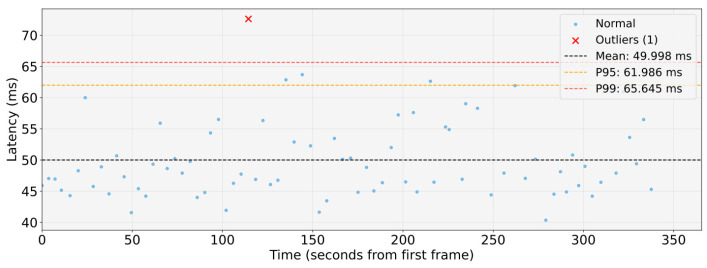
End-to-end timing analysis of the object-detection perception-planning pipeline.

**Figure 16:**
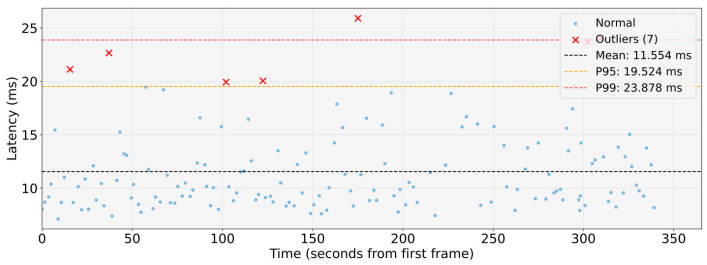
End-to-end timing analysis of the obstacle-detection perception-planning pipeline.

Figure 14, Figure 15 and Figure 16 show graphs illustrating collected data and statistical analysis performed on end-to-end latency measurements.

Both results of the WCET and of the end-to-end analysis corroborate the real-time capabilities of the proposed architecture. In the test-vehicle scenario, the maximum reaction time is dominated by the object-detection and lane-detection pipelines, yet the perception–planning chain still remains within the 100 ms end-to-end time budget, which, as discussed above, meets the real-time constraints for human-level safety. Assuming a cruising speed of 0.5m/s, the above values $WCET_{\mathrm{tot}}$ imply that a new frame can be processed by the object-detection pipeline approximately every 0.0472m of the distance travelled, whereas the obstacle-detection pipeline allows an update approximately every 0.0124m. These update rates are sufficient to ensure real-time operation in scenario analysed, which target autonomous mobile robotic platforms, including the most demanding perception–planning pipelines.

**Real-time performance of perception-planning pipelines**

The proposed WCET and end-to-end timing analysis suggest that the architecture is suitable for real-time autonomous mobile robotics in terms of latency. The runtime performance of the presented perception planning pipelines is discussed. For object and obstacle detection, a qualitative assessment was providedbased on integrated executions of the full ROS 2 architecture on the target platform: in both cases, the algorithms run reliably at the average end-to-end latencies reported in the analysis above, producing stable outputs with no evident compromises (e.g., no backlog accumulation, stalls, or responsiveness degradation). Regarding lane keeping, a quantitative evaluation computed from the *rosbag* recordings, collected during experimental trials in our test environment, was additionally reported. The lateral tracking error was calculated with respect to the lane centreline and standard accuracy metrics were derived. Table 8 reports the metrics computed from the collected data, the MAE (Mean Absolute Error) and the RMSE (Root Mean Square Error), respectively. Furthermore, from the rosbag logs collected during the experimental trials, we could derive the effective pipeline update rate, showing an average lane-keeping reference update frequency of 22.4 Hz (≈44ms period), which is consistent with the results presented above and provides additional experimental validation of the end-to-end timing analysis.

## 7. Conclusions

This work presented a ROS 2-based architecture for autonomous vehicles, validated through experiments on a 1:10 scale vehicle model operating in a realistic, city-like testing environment. The proposed framework demonstrated effective modularity, scalability, and maintainability across the Perception, Planning, and Control layers, confirming its suitability for autonomous ground mobile robots and embedded robotic systems.

The architecture integrates three functional layers developed and tested on two different hardware platforms, demonstrating portability and adaptability to heterogeneous computing resources. A rule-based decision tree (RBDT) combined with a finite-state machine (FSM) governs the Planning layer, ensuring interpretable and reliable manoeuvre execution. Synchronisation between asynchronous data streams is achieved through lightweight upsampling and downsampling strategies, complemented by an event-driven Decision-Making Package (DMP) based on a zero-order hold mechanism. This design preserves temporal consistency without introducing unnecessary computational overhead, making it well suited for real-time embedded applications.

Experimental validation confirmed that the system meets real-time requirements along the perception–planning pipeline, maintaining bounded execution times and predictable response under variable workloads. The results highlight the architecture’s ability to achieve deterministic performance while preserving flexibility and modularity.

The experimental results confirm that the proposed architecture satisfies the requirements of autonomous vehicles and, more broadly, of autonomous ground mobile robots, with particular emphasis on scalability, a critical aspect in this domain.

In real-world deployments, the proposed architecture lends itself to a range of use cases spanning from educational/prototyping setups to robot-scale autonomous mobile platforms. The latter represent a particularly relevant target domain, as mobile robots are often equipped with resource-constrained on-board compute platforms (limited CPU/GPU and memory resources), and therefore benefit from an architecture designed to be modular, scalable, and efficient while still supporting complete perception–planning pipelines under practical real-time constraints.

## 8. Future Works

Future research will focus on migrating the current Control Layer implementation from a bare-metal STM32 platform to a micro-ROS–based architecture. This transition will enable the embedded control components to act as ROS 2 nodes exposing services and actions within the DDS-XRCE communication framework, thereby achieving a fully service-oriented Architecture at the microcontroller level. Using micro-ROS and its RTOS backends, it will be possible to investigate deterministic scheduling strategies and Quality-of-Service configurations tailored to control-critical interactions. This approach is expected to improve the interoperability, scalability, and modularity of the system while aligning with recent efforts toward service-oriented robotic software architectures. Therefore, ongoing and future work will focus on evaluating performance trade-offs in terms of latency, resource utilisation, and reliability, establishing guidelines for the deployment of service-oriented robotic control on resource-constrained embedded platforms.

## Figures and Tables

**Table 1:** Sensors Domain and Update Frequencies by System Layer.

Layer	Sensor	Update Frequency
Perception	Cameras	low-to-medium range (e.g., 20 Hz)
	LiDAR	low-to-medium range (e.g., 10 Hz)
	IMU	low-to-medium range (e.g., 20 Hz)
	GPS (indoor)	typically low (e.g., 5 Hz)
Control	Wheel Encoder	typically high (e.g., 100 Hz)
	IMU	typically high (e.g., 50 Hz)

**Table 2:** Perception Topics and custom messages.

Topic	Custom Message Type	Description
*\detected_lane*	LaneDetected	Contains the lane points detected
\detected_obj	ObjectDetected	Contains the type of object detected (e.g., sign, pedestrian) and its distance
\global_pos	GlobalPos	Contains all the coordinates representing the car’s position and orientation
\detected_obst	ObstacleDetected	Contains the type of obstacle detected (e.g., car, sign, pedestrian) as well as its direction and distance

**Table 3:** Input Feature Set for the Rule-Based Decision Tree.

Feature	Type	Range	Description
Obstacle	Boolean	{0, 1}	Presence of obstacle within safety margin
Maneuvering	Boolean	{0, 1}	Active maneuver flag
Sign	Int	{−1, 11}	Recognized traffic sign ID from vision-based classifier
StopLine	Boolean	{0, 1}	Proximity to intersection stop line
PathPlanning	Int	{0, 3}	Global path context
IntersectionSignFlag	Boolean	{0, 1}	Presence of intersection-related signage
DoOvertake	Boolean	{0, 1}	Feasibility of overtaking based on adjacent lane occupancy

**Table 4:** Sensor equipment of the 1:10 scale Vehicle Platform used.

Sensor	Model	Description
Camera	Sony IMX219 (Sony Corporation, Tokyo, Japan)	Wide angle RGB camera module, for vision related algorithms (e.g.,: lane detection, object detection, …)
IMU	Bosch BNO055 (Bosch Sensortec GmbH, Reutlingen, Germany)	9DOF IMU module
Encoder	Generic Magnetic encoder	Generic magnetic encoder mounted on traction motor shaft (16 PPR resolution)
2D LiDAR	LDROBOT LD19 (LDROBOT Co., Ltd., Shenzhen, China)	2D LiDAR for obstacle detection and environment scanning
GPS(indoor)	Qorvo MDEK1001 (Qorvo, Inc., Greensboro, NC, USA)	UWB development kit for localization infrastructure in the test environment.

**Table 5:** Actuators equipment of the 1:10 scale Vehicle Platform used.

Actuator	Model	Description
Traction Motor	Generic DC Motor	Brushed 12V DC motor with integrated gearbox (1:5.2 reduction ratio) for wheel traction.
Steer Motor	MG996R servomotor (TowerPro, Shenzhen, China)	Servomotor for steering control.

**Table 6:** Measured execution times for key tasks in the perception-planning pipeline.

Task	Mean Exec. Time [ms]	P95 Exec. Time [ms]	Max Exec. Time [ms]
CAM1 frame preprocessing	8.318	13.484	23.480
CAM2 frame preprocessing	7.412	11.733	14.717
Lane Detection algorithm	33.831	39.245	52.102
Object Detection algorithm	28.853	33.243	49.458
Obstacle Detection algorithm	0.375	0.875	4.436
Decision Making	6.675	11.082	15.869
State Machine	0.647	2.194	5.560

**Table 7:** End-to-end timing analysis among the analysed perception-planning pipelines.

**End-to-end timing analysis: Lane detection pipeline**	
Mean Exec.Time [ms]	43.221
P95 Exec.Time [ms]	49.538
Max Exec.Time [ms]	62.061
**End-to-end timing analysis: Object detection pipeline**	
Mean Exec.Time [ms]	49.998
P95 Exec.Time [ms]	61.986
Max Exec.Time [ms]	72.616
**End-to-end timing analysis: Obstacle detection pipeline**	
Mean Exec.Time [ms]	11.554
P95 Exec.Time [ms]	19.524
Max Exec.Time [ms]	25.909

**Table 8:** Lane-keeping performance metrics computed from rosbag logs (lateral error w.r.t. lane centerline).

Lane-Keeping Tracking Metrics	
MAE [m]	0.0282
RMSE [m]	0.0350

## Data Availability

The original contributions presented in this study are included in the article. Further inquiries can be directed to the corresponding author.

## Abbreviations

The following abbreviations are used in this manuscript:

AV	Autonomous Vehicle
ROS	Robot Operating System
SOA	Service-Oriented Architecture
DDS	Data Distribution System
QoS	Quality of Service
RBDT	Rule Based Decision Tree
FSM	Finite State Machine
DM	Decision Making
DMP	Decision Making Package
ECU	Electronic Control Units
GPS	Global Positioning System
LiDAR	Light Detection And Ranging
IMU	Inertial Measurement Unit
WCET	Worst Case Execution Time
